# Avibactam potentiated the activity of both ceftazidime and aztreonam against *S. maltophilia* clinical isolates in vitro

**DOI:** 10.1186/s12866-021-02108-2

**Published:** 2021-02-22

**Authors:** Qiuxia Lin, Hua Zou, Xian Chen, Menglu Wu, Deyu Ma, Hanbing Yu, Siqiang Niu, Shifeng Huang

**Affiliations:** 1 Department of Clinical Laboratory Medicine, The First Affiliated Hospital of Chongqing Medical University, No. 1 Friendship Road, Yuzhong District, Chongqing, 400016 China; 2 Department of Clinical Laboratory Medicine, Chongqing Health Center for Women And Children, 120 Longshan Road, Yubei District, Chongqing, 400016 China; 3 Department of Clinical Laboratory, The Affiliated Hospital of Qingdao University, No. 59, Haier Road, Laoshan District, Qingdao, 266061 China; 4 Department of Clinical Laboratory, Qingdao Women’s and Children’s Hospital, No.6, Tongfu Road, Shibei District, Qingdao, 266061 China

**Keywords:** *Stenotrophomonas maltophilia*, Ceftazidime-avibactam, Aztreonam-avibactam, Minimum inhibitory concentrations

## Abstract

**Background:**

Treatment options for *Stenotrophomonas maltophilia (S. maltophilia)* infections were limited. We assessed the efficacy of ceftazidime (CAZ), ceftazidime-avibactam (CAZ-AVI), aztreonam (ATM), and aztreonam-avibactam (ATM-AVI) against a selection of 76 *S. maltophilia* out of the 1179 strains isolated from the First Affiliated Hospital of Chongqing Medical University during 2011–2018.

**Methods:**

We investigated the antimicrobial resistance profiles of the 1179 *S. maltophilia* clinical isolates from the first affiliated hospital of Chongqing Medical University during 2011–2018, a collection of 76 isolates were selected for further study of microbiological characterization. Minimum inhibitory concentrations (MICs) of CAZ, CAZ-AVI, ATM and ATM-AVI were determined via the broth microdilution method. We deemed that CAZ-AVI or ATM-AVI was more active in vitro than CAZ or ATM alone when CAZ-AVI or ATM-AVI led to a category change from “Resistant” or “Intermediate” with CAZ or ATM alone to “Susceptible” with CAZ-AVI or ATM-AVI, or if the MIC of CAZ-AVI or ATM-AVI was at least 4-fold lower than the MIC of CAZ or ATM alone.

**Results:**

For the 76 clinical isolates included in the study, MICs of CAZ, ATM, CAZ-AVI and ATM-AVI ranged from 0.03–64, 1–1024, 0.016–64, and 0.06–64 μg/mL, respectively. In combined therapy, AVI was active at restoring the activity of 48.48% (16/33) and 89.71% (61/68) of *S. maltophilia* to CAZ and ATM, respectively. Furthermore, CAZ-AVI showed better results in terms of the proportion of susceptible isolates (77.63% vs. 56.58%, *P* < 0.001), and MIC50 (2 μg/mL vs. 8 μg/mL, *P* < 0.05) when compared to CAZ. According to our definition, CAZ-AVI was more active in vitro than CAZ alone for 81.58% (62/76) of the isolates. Similarly, ATM-AVI also showed better results in terms of the proportion of susceptible isolates (90.79% vs.10.53%, *P* < 0.001) and MIC50 (2 μg/mL vs. 64 μg/mL, *P* < 0.001) when compared to ATM. According to our definition, ATM-AVI was also more active in vitro than ATM alone for 94.74% (72/76) of the isolates.

**Conclusions:**

AVI potentiated the activity of both CAZ and ATM against *S. maltophilia* clinical isolates in vitro. We demonstrated that CAZ-AVI and ATM-AVI are both useful therapeutic options to treat infections caused by *S. maltophilia.*

## Background

*Stenotrophomonas maltophilia* (*S. maltophilia*) has emerged as an important cause of nosocomial infections in immunocompromised hosts. In patients with cystic fibrosis (CF), *S. maltophilia* is known for colonizing the airways and causing chronic infections [1, 2]. Although *S. maltophilia* is primarily associated with respiratory tract infections, this pathogen can cause a wide range of clinical syndromes, including catheter-associated bloodstream infections, and skin and soft tissue infections [2–4]. Furthermore, it has emerged as an important nosocomial pathogen associated with crude mortality rates ranging from 14 to 69% in patients with bacteremia [5, 6]. *S. maltophilia* is recognized by the World Health Organization as one of the leading MDR organisms for which disease prevention and treatment strategies must be developed [7]. More frustratingly, *S. maltophilia* is intrinsically resistant to different classes of antibiotics used in clinical practices, which was mediated by the expression of aminoglycoside-modifying enzymes, qnrB-like quinolone-resistant determinants, multidrug efflux pumps, and two β-lactamases (L1 and L2) [1, 8, 9]. These characteristics, together with its ability to adapt to environmental changes, contribute to the difficulty in effectively managing *S. maltophilia* infections.

Ceftazidime (CAZ), Levofloxacin (LVX), minocycline (MH), and trimethoprim-sulfamethoxazole (SXT) have been used as treatment options for *S. maltophilia* infections, but unfortunately, their susceptibility against *S. maltophilia* is declining [10].

*S. maltophilia* isolates naturally produce two β-lactamases (L1 and L2). L1 is a class B3 metallo-β-lactamase (MBL) that hydrolyzes carbapenems and other β-lactams, with the important exception of the monobactam aztreonam (ATM), and is resistant to all clinically available β-lactamase inhibitors [1, 8, 9]. L2 is a class A cephalosporinase that confers resistance to extended-spectrum cephalosporins and ATM but can be inhibited by commercially available serine-β-lactamase inhibitors such as clavulanic acid and avibactam (AVI) [1, 2, 11, 12]. AVI is a non­-β-­lactam, β-­lactamase inhibitor without intrinsic antibacterial activity, but offers a broader β-lactamase inhibition profile compared with any other recently used serine β-lactamase inhibitors [13]. Although *S. maltophilia* isolates naturally produce two β-lactamases (L1 and L2), the inhibitory activities of CAZ and ATM against L2-producing *S. maltophilia* isolates might theoretically be re-established by AVI combination; furthermore, given the potential bactericidal activity of ATM against L1-producing *S. maltophilia* isolates, ATM-AVI might be useful to treat infections with both L1- and L2-producers. On the other hand, two recent studies from France and Japan demonstrated that CAZ-AVI is not active against *S. maltophilia* [14, 15]. However, another report from France showed that while one third of the *S. maltophilia* isolates studied remained resistant to CAZ-AVI, 30% showed low MICs (< 1 μg/mL) [16], highlighting the potential benefit of CAZ-AVI against *S. maltophilia*. Therefore, for better decision making of the clinical management of *S. maltophilia* infections, additional epidemiology and resistance testing of *S. maltophilia* isolates from other countries or regions worldwide are desperately needed. In addition, alternative therapeutic strategy for *S. maltophilia* is in urgent need. So far, there are few data available in China describing the in vitro activities of CAZ-AVI and ATM-AVI against *S. maltophilia* clinical isolates. We conducted an observational study to evaluate the in vitro antimicrobial activities of CAZ-AVI and ATM-AVI against recent *S. maltophilia* clinical isolates in our hospital. In this paper, we demonstrated that CAZ-AVI combination inhibited about half (16/33, 48.48%) of the CAZ-non-susceptible (CAZ-NS) *S. maltophilia* isolates, while ATM-AVI combination inhibited most (61/68, 89.71%) of the ATM-non-susceptible (ATM-NS) *S. maltophilia* strains.

## Results

### Microbiological characteristics and antimicrobial susceptibility profiles of *S. maltophilia* isolates

In total, 1179 non-repetitive *S. maltophilia* strains were isolated during the study period, among which the predominant sample origins were sputum (73.0%), followed by secretion from various body sites such as secretions from surgical incisions, burns, or conjunctival sacs (7.0%) and urine (5.0%). Department distribution analysis showed that ICUs (39.0%), medical wards (36.0%), and surgical wards (25.0%) contributed the majority of *S. maltophilia* isolates. With regard to the antimicrobial susceptibility profiles of the *S. maltophilia* isolates, its non-susceptible rates to LVX, MH, and SXT were respectively 8.23, 3.14, and 6.28% (Table 1).

**Table 1:** Antimicrobial susceptibility test results of 1179 *S.maltophilia* clinical isolates

Antibiotics	Sensitive	Intermediate	Resistant	Non-susceptible rate
Levofloxacin (LVX)	1082	16	81	8.23%
Minocyline (MH)	1142	25	12	3.14%
Sulfamethoxazole (SXT)	1105	21	53	6.28%

### Inhibitory activities of CAZ-AVI and ATM-AVI against *S. maltophilia* isolates

To assess the potential efficacy of CAZ-AVI and ATM-AVI against *S. maltophilia* isolates, we have tested these combinations in vitro on a collection of 76 non-repetitive isolates available for microbiological characterization. The majority of tested isolates (60/76, 78.95%) were resistant to at least one of the following three antibiotics, namely LVX, MH, and SXT. While the remaining 16 isolates were sensitive to LVX, MH and SXT. The in vitro antimicrobial susceptibilities of CAZ, CAZ-AVI, ATM, and ATM-AVI against these isolates were determined using the Clinical and Laboratory Standards Institute (CLSI) broth microdilution method. The MIC of CAZ alone was <=8 μg/mL for 43 of 76 *S. maltophilia* isolates (56.58%), and the MIC of CAZ-AVI was <=8/4 μg/mL for 59 of 76 isolates (77.63%) (Table 2). According to our definition, CAZ-AVI was more active in vitro than CAZ alone for 81.58% (62/76) of the isolates. For the 16 out of the 33 (48.48%) *S. maltophilia* isolates that were CAZ-NS, the combination of AVI restored their in vitro activity to CAZ (9 isolates from resistant to sensitive, and 7 isolates from intermediate to sensitive). AVI addition also reduced the CAZ MIC50 of the 76 *S. maltophilia* isolates from 8 to 2 μg/mL (Table 3). In all, the MIC of ATM alone was <=8 μg/mL for 8 of the 76 *S. maltophilia* isolates (10.53%), and the MIC of ATM-AVI was <=8/4 μg/mL for 69 of 76 isolates (90.79%) (Table 2). ATM-AVI was more active in vitro than ATM alone for 94.74% (72/76) of the isolates. Notably, for 61 out of the 68 (89.71%) *S. maltophilia* isolates that were ATM-NS, the addition of AVI restored in vitro activity to ATM (60 isolates from resistant to sensitive, and 1 isolate from intermediate to sensitive). AVI addition also reduced the ATM MIC50 of the 76 *S. maltophilia* isolates from 64 to 2 μg/mL (Table 3).

**Table 2:** MICs of CAZ and ATM in 76 *S. maltophilia* isolates: alone or in combination with 4 μg/mL AVI

antibiotics	MIC (μg/mL)																
	0.016	0.03	0.06	0.12	0.25	0.5	1	2	4	8	16	32	64	128	256	512	1024
ATM	0	0	0	0	0	0	1	1	1	5	1	2	59	2	0	3	1
ATM + AVI	0	0	1	2	0	4	18	35	5	4	2	3	2	0	0	0	0
CAZ	0	1	0	0	0	1	3	8	12	18	9	2	22	0	0	0	0
CAZ + AVI	1	0	0	1	2	3	8	28	9	7	5	2	10	0	0	0	0

*MIC* Minimum inhibitory concentrations; *CAZ* ceftazidime; *ATM* aztreonam; *AVI* avibactam

**Table 3:** MIC50s, MIC90s, and ranges of MICs of CAZ-AVI and ATM-AVI for 76 *S. maltophilia* isolates from clinical specimens^a^

Drug resistance phenotype (s) ^b^ (no. of isolates)	CAZ			CAZ + AVI			CAZ-AVI MIC50 reduction (fold)	ATM			ATM + AVI			ATM-AVI MIC50 reduction (fold)
	MIC50	MIC90	range	MIC50	MIC90	range		MIC50	MIC90	range	MIC50	MIC90	range	
Total(76)	8	64	0.03–64	2	64	0.016–64	4	64	64	1–1024	2	8	0.06–64	32
MH-NS^c^ or LVX-NS or SXT-NS (60)	16	64	0.03–64	4	16	0.016–64	4	64	64	1–1024	2	8	0.06–64	32
MH-S^d^ and LEV-S and SXT-S (16)	4	8	1–16	2	2	0.25–8	2	64	64	64	2	2	0.5–64	32
MDR (2)	NA^e^	NA	> = 64	NA	NA	16- > 64	NA	NA	NA	64	NA	NA	2–8	NA

^a^MICs are expressed in μg/mL

^b^CAZ ceftazidime, ATM aztreonam, AVI avibactam, MDR multidrug resistant. Multidrug-resistant isolates were defined as isolates demonstrating resistance to at least one antimicrobial agent from three or more different classes

^c^NS not susceptible, ^d^S susceptible, ^e^NA not applicable. MIC50s and MIC90s are not presented for groups of fewer than 6 isolates

The results of susceptibility testing comparing CAZ, CAZ-AVI, ATM and ATM-AVI, are shown in (Table 3). These agents showed a wide range of activity against *S. maltophilia*. In detail, the ranges of MICs of CAZ and ATM for the 76 clinical *S. maltophilia* isolates were 0.03 to 64 and 1 to 1024 μg/mL, respectively, while those of CAZ-AVI and ATM-AVI for the same *S. maltophilia* isolates were 0.016 to 64 and 0.06 to 64 μg/mL, respectively (Table 3). In this study, AVI potentiated the activity of ATM against most of the *S. maltophilia* clinical isolates tested in vitro. Meanwhile, AVI also enhanced the activity of CAZ against most of those isolates tested in vitro. For *S. maltophilia* isolates that were non-susceptible to LVX, MH, and/or SXT (78.95%, 60 of 76), the addition of 4 μg/mL AVI greatly increased the activity of CAZ against most isolates (4-fold MIC50 reduction) and the addition of 4 μg/mL AVI also significantly increased the activity of ATM against most isolates (32-fold MIC50 reduction) (Table 3). AVI did not restore the activity of CAZ against the two multidrug-resistant (MDR) *S. maltophilia* isolates, even though AVI reduced one MDR isolate’s MIC somewhat (4-fold MIC reduction) (Table 3). However, AVI did restore the activity of ATM against the two MDR *S. maltophilia* isolates with obvious MIC reduction of 32- or 8-fold. (Table 3).

## Discussion

*S. maltophilia* infections pose a major challenge for clinicians because of limited therapeutic options. For the 76 clinical isolates included in the present study, both CAZ-AVI and ATM-AVI exerted promising results in terms of the proportion of susceptible isolates and MIC50. Furthermore, while ATM-AVI was more active in vitro than ATM alone for 94.74% of the isolates, CAZ-AVI was more active in vitro than CAZ alone for 81.58% of the isolates. However, it is noteworthy that CAZ-AVI resistance was found in 12 *S. maltophilia* strains isolated from patients with no history of previous CAZ-AVI-based treatment, moreover, 5 *S. maltophilia* strains isolated from patients without previous ATM-AVI exposure demonstrated in vitro resistance to ATM-AVI, indicating that incidence of CAZ-AVI and ATM-AVI resistance could emerge in *S. maltophilia* strains without previous antimicrobial exposure. As expected, the strains that were resistant to CAZ-AVI or ATM-AVI combinations had also been resistant to CAZ or ATM alone**.**

Notably, although AVI did not restore the activity of CAZ against the two MDR *S. maltophilia* isolates, it did restore the activity of ATM against the two MDR *S. maltophilia* strains with significant MIC reductions of 32- and 8-fold respectively. The poor activity of CAZ-AVI against MDR *S. maltophilia* isolates was in accordance with a previous study by Lindsay J. Caverly et al. [17], who demonstrated that the activity of CAZ-AVI was poor against most MDR/XDR *S. maltophilia* strains. Recently, the efficacy of CAZ-AVI (2.5 g i.v. every 8 h) in combination with ATM (2 g i.v. every 8 h) for 48 days was demonstrated for a young renal transplant patient with *S. maltophilia* resistant to SXT, meropenem and CAZ [12]. The synergy among CAZ, ATM and AVI is encouraging and deserves further exploration.

When compared with CAZ-AVI, ATM-AVI exhibited obviously superior inhibitory activity, inhibiting the growth of 89.71% of the ATM-NS isolates (61/68) (Table 2). Nevertheless, although the results of some prior studies [14, 15] didn’t show CAZ-AVI to be active against *S. maltophilia*, in our research, CAZ-AVI has been demonstrated to inhibit the growth of about half of the CAZ-NS isolates (48.48%, 16/33). As *S. maltophilia* isolates naturally produce L1 and/or L2 β-lactamases: while L2 is a class A cephalosporinase that confers resistance to CAZ but can be inhibited by AVI [1, 2, 11, 12], L1 is an MBL that can hydrolyze CAZ but can’t be inhibited by AVI. Thus, while the L1-producers can’t be killed by CAZ-AVI, the growth of the L2-producers might be inhibited by CAZ-AVI. Thus the variable results from different studies may have been due to the different β-lactamases that different *S. maltophilia* isolates from various districts produced.

In this study, we likewise proposed the possible emergence of both CAZ-AVI non-susceptible and ATM-AVI non-susceptible *S. maltophilia* strains isolated from patients without previous antimicrobial exposure to CAZ-AVI and ATM-AVI, which are consistent with our previous findings that demonstrated CAZ-AVI resistance in the carbapenem-resistant *Enterobacteriaceae* (CRE) bacteremia isolates from patients with no history of previous CAZ-AVI exposure [18].

The current study has several limitations. First of all, we only evaluated the activity of CAZ, ATM, CAZ-AVI and ATM-AVI without exploring the resistance mechanisms for both CAZ-AVI and ATM-AVI non-susceptibilities in our *S. maltophilia* isolates. Secondly, this was a single-center retrospective study with relatively small sample size conducted in Chongqing.

## Conclusions

In summary, ATM-AVI showed the most potent in vitro activity among the other related agents, including CAZ, ATM and CAZ-AVI, against *S. maltophilia* isolates. The excellent in vitro activity of CAZ-AVI or ATM-AVI against *S. maltophilia* isolates in our hospital supports further evaluation of CAZ-AVI or ATM-AVI in clinical studies against *S. maltophilia* infections. CAZ-AVI or ATM-AVI might turn out to be useful therapeutic options to treat infections caused by *S. maltophilia*.

## Methods

### Bacterial strains

We collected a total of 76 non-repetitive, recent nosocomial *S. maltophilia* strains between 2014 and 2018 in the First Affiliated Hospital of Chongqing Medical University. All the isolates were identified at the species level by the VITEK MS (bioMérieux, MO, USA) system, and routine antimicrobial susceptibility testing was performed using the disk diffusion (for LVX, MH, and SXT) testing methods. All the *S. maltophilia* colonization and infection cases (1179) with complete medical records during 2011–2018 were investigated for clinical and antimicrobial resistance profiles, among which 76 recent isolates during 2014–2018 were selected for further microbiological characterization. In order to systemically include a range of different resistance profiles, we collected 60 *S. maltophilia* strains resistant to at least one of the three clinically available drugs (LVX, MH, and SXT), and 16 isolates sensitive to all the three antibiotics as comparison.

### Antibiotics and in vitro antimicrobial susceptibility testing

A collection of 76 non-repetitive *S. maltophilia* isolates during 2014–2018 were recovered for CAZ, CAZ-AVI, ATM, and ATM-AVI susceptibility tests. MIC values of CAZ, CAZ-AVI, ATM, and ATM-AVI were determined by broth microdilution method as described by the Clinical and Laboratory Standards Institute M07-Ed11 (2018) [19]. MICs were firstly tested in duplicate, when the duplicated results were equal, MICs were determined, whereas for cases where replicates were different, a second test in duplicate was further conducted, and the most frequent MIC value was chosen as the final MIC value. MICs of CAZ and ATM alone and in combination with AVI at a fixed concentration of 4 μg/mL were measured according to CLSI criteria of *Enterobacteriaceae* [20]. Antibiotic solutions for susceptibility testing were prepared fresh. All samples were incubated at 35 °C for 18 to 20 h prior to MIC determination [19]. MICs of CAZ were interpreted on breakpoints for *S. maltophilia* (8/16/32 μg/mL). MICs of ATM and comparator agents were interpreted on breakpoints for *Pseudomonas aeruginosa* (8/16/32 μg/mL) according to CLSI criteria in M100-Ed29, 2019 [20]. MICs of CAZ > =32 μg/mL, CAZ-AVI > =32/4 μg/mL, ATM > =32 μg/mL, and ATM-AVI > =32/4 μg/mL were considered resistant. We deemed that CAZ-AVI or ATM-AVI was more active in vitro than CAZ or ATM alone when CAZ-AVI or ATM-AVI led to a category change from “Resistant” or “Intermediate” with CAZ or ATM alone to “Susceptible” with CAZ-AVI or ATM-AVI, or if the MIC of CAZ-AVI or ATM-AVI was at least 4-fold lower than the MIC of CAZ or ATM alone.

### Statistical analysis

Distributions of continuous variables were compared between two groups using Student’s t-test (normally distributed variables) and Wilcoxon rank-sum test (non-normally distributed variables) as appropriate. For all calculations, *P* < 0.05 was considered statistically significant. All analyses were performed using the SPSS v.25.0 software (SPSS Inc., IL, USA).

## Data Availability

The datasets used and/or analysed during the current study are available from the corresponding author on reasonable request.

## Abbreviations

*S. maltophilia*: Stenotrophomonas maltophilia
CAZ: Ceftazidime
ATM: Aztreonam
AVI: Avibactam
CAZ-AVI: Ceftazidime-avibactam
ATM-AVI: Aztreonam-avibactam
MICs: Minimum inhibitory concentrations
LVX: Levofloxacin
MH: Minocycline
SXT: Trimethoprim-sulfamethoxazole
NS: Non-susceptible
